# Extracellular Vesicles (EVs) from Lung Adenocarcinoma Cells Promote Human Umbilical Vein Endothelial Cell (HUVEC) Angiogenesis through Yes Kinase-associated Protein (YAP) Transport

**DOI:** 10.7150/ijbs.31605

**Published:** 2019-08-07

**Authors:** Ying Wang, Liyang Dong, Hai Zhong, Linfei Yang, Qian Li, Chuan Su, Wei Gu, Yingying Qian

**Affiliations:** 1 Department of Respiration, Nanjing First Hospital, Nanjing Medical University, 68 Changle Road, Nanjing, Jiangsu 210006, People' s Republic of China; 2 Department of Nuclear Medicine, The Affiliated Hospital of Jiangsu University, 438 Jiefang Road, Zhenjiang, Jiangsu 212002, People' s Republic of China; 3 Department of Respiration, Jiangning Hospital, Nanjing Medical University, 168 Gushan Road, Nanjing, Jiangsu 211101, People' s Republic of China; 4 Jiangsu Key Laboratory of Pathogen Biology, Department of Pathogen Biology and Immunology, Center for Global Health, Nanjing Medical University, Nanjing, Jiangsu 211166, People's Republic of China

**Keywords:** YAP, lung adenocarcinoma, H1975 cell, EVs, angiogenesis

## Abstract

Yes kinase-associated protein (YAP) plays an important role in angiogenesis and can promote the occurrence and development of many tumor types. However, whether YAP affects tumor angiogenesis in lung cancer, and its potential mechanism in lung cancer, are unknown. In this study, we explored the role of YAP in the angiogenesis of lung adenocarcinoma, and further illustrated its possible mechanism. The expression levels of YAP and the vascular endothelial marker protein CD31 were examined by immunohistochemistry and immunofluorescence in human lung adenocarcinoma tissues, revealing a possible positive correlation between YAP and CD31 in lung adenocarcinoma. The results of the western blotting (WB) of Human Umbilical Vein Endothelial Cells (HUVECs) after coculture with lung adenocarcinoma H1975 cells, H1975 cell-supernatants and H1975-derived EVs showed that YAP derived from H1975 cells can enter HUVECs via EVs. These results were confirmed by immunofluorescence. Finally, we generated H1975 low-YAP expression cells by transfecting the cells with a shYAP lentivirus, and confirmed that the low expression of YAP in H1975 cells inhibits HUVEC angiogenesis by reducing the amount of YAP that enters HUVECs. We found, for the first time, that YAP promotes angiogenesis in lung adenocarcinoma via EVs, at least partially. Our work may provide a promising method for lung cancer treatment by targeting angiogenesis in the future.

## Introduction

Lung cancer is the leading cause of cancer-associated death worldwide 1. Some 80-85% of lung cancers are non-small cell lung cancer (NSCLC), which includes lung adenocarcinoma (LUAD), lung squamous cell carcinoma (LSCC), and large-cell carcinoma 2, with the first being the most common type. The incidence of LUAD is increasing worldwide 3, and it is clear that cancer progression is the result of the interaction of various factors in the tumor microenvironment. Studies have indicated that the formation of new blood vessels in the tumour microenvironment is a key step in the growth of solid tumors and is a prerequisite for tumor invasion and metastasis 4. Thus, a better understanding of the mechanism of tumor angiogenesis may provide new targets for the treatment of LUAD.

The Hippo signaling pathway is a highly conservative signal transduction pathway that was found to regulate organ development and maintain tissue stability in the genus *Drosophila* in recent years 5. Yes kinase-associated protein (YAP) is the main downstream effector of the Hippo signaling pathway and can promote cell proliferation, inhibit apoptosis, and maintain stem cell phenotypes 6. Recent studies have found that YAP plays an important role in angiogenesis and confirmed that, as a potential oncogene, YAP overexpression or overactivation promotes the occurrence and development of various types of tumors 7-9. Marti et al 10 found that, in vitro or in vivo, upregulated YAP expression in tumor cells promotes the angiogenesis of vascular endothelial cells in cholangiocarcinoma. The interference of YAP expression in a gastric cancer mouse model inhibits angiogenesis 11. Research has found that over 60% of NSCLC patients have abnormal activation of YAP, and YAP overexpression contributes to poor prognosis in NSCLC 12. These results suggest that YAP may be involved in the progression of lung cancer. However, whether YAP plays a role in tumor angiogenesis in lung cancer, remains unknown.

Extracellular Vesicles (EVs) have been of great interest as effective carriers that are involved in intercellular communication 13, 14. EVs are membrane-surrounded structures that are released by cells and contain complex cargoes, including nucleic acids, proteins, and lipids 15. EVs have been purified from nearly all mammalian cell types, including numerous cancer cell lines 16-18. Cancer-derived EVs regulate many aspects of tumorigenesis, including tumor angiogenesis 19, the recruitment of cancer-associated fibroblasts 20, metastatic potential and the evasion of immune destruction 21. Moreover, tumor EVs can be used as carriers to transfer parent cell material to recipient cells. For example, EVs derived from tumor cells have been shown to transfer activated EGFR to endothelial cells 18. However, whether YAP is a mediator that is transferred from lung cancer cells to endothelial cells by EVs, thereby promoting endothelial cell angiogenesis is unknown.

In this study, we found that functional YAP is overexpressed in LUAD cells and LUAD cells could secrete YAP via EVs. LUAD cell derived EVs promoted the tubulogenesis of endothelial cells *in vitro* and this ability is related to the content of YAP in EVs. Our study may illuminate a new mechanism of lung cancer angiogenesis.

## Materials and Methods

### Patients and tissue specimens

The specimens of lung adenocarcinoma and normal lung tissue were obtained from surgical specimens from the Nanjing First Hospital from January to December 2016. Patients who underwent preoperative radiotherapy or chemotherapy were excluded. All the patients or their guardians signed informed consent forms before they participated in the study. The study was conducted in accordance with the principles of the Declaration of Helsinki, and the protocol was approved by the Ethics Committee of Nanjing First Hospital.

### Immunohistochemistry

The specimens were fixed with 10% formalin for 72 hours, and then were paraffin-embedded and cut into sections with a 4-µm thickness. Paraffin-embedded tissue sections were stained with anti-YAP antibody (Cell Signaling Technology Inc., Danvers, MA) according to the manufacturer's instructions. The sections were incubated with a primary antibody at 4°C overnight and were stained with an appropriate secondary antibody conjugated with horseradish peroxidase. Next, the sections were stained with the chromogen diaminobenzidine, washed with PBS, counterstained with hematoxylin and dehydrated. All the biopsies were independently examined by two pathologists. Five observations of each sample were made to evaluate the specimens.

### Cell culture

The lung cancer cell line H1975 and normal human bronchial epithelial cell line BEAS-2B were purchased from the American Type Culture Collection (Manassas, VA). Human umbilical vein endothelial cells (HUVECs) were donated by the Department of Cardiology, Nanjing First Hospital. All the cell lines were cultured in DMEM (Gibco, Carlsbad, CA) supplemented with 10% FBS (Gibco) and 1% pen/strep (Gibco) under conditions of 5% CO_2_ and 37 °C.

### Real-time PCR

RNA was isolated from cells with TRIzol reagent (Invitrogen, Carlsbad, CA) according to the manufacturer's instructions. The isolated RNA was reverse transcribed into cDNA using an All-in-one^TM^-first-cDNA synthesis kit (Complex Energy Gene, Hangzhou, China). Quantitative real-time PCR was performed with a CFX96™ Real-Time system (Bio-Rad, Hercules, CA) and SYBR Green PCR master mix (Applied Biosystems). The fold change in the gene expression was calculated with the 2^-ΔΔCt^ method, and three replicates were performed for each cDNA sample. GAPDH was used as an internal reference. The YAP mRNA level is expressed as the fold change relative to the expression level in HUVECs (as control). The sequences of the primers were as follows: YAP forward, 5′-TAC GAT ACA AGG CTG TTA GAG AG-3′ and reverse, 5′-TTG AGA TGC ATG CTT TGC ATAC-3′; GAPDH forward, 5′-GAA GGT GAA GGT CGG AGT C-3′ and reverse, 5′-GAA GAT GGT GAT GGG ATT TC-3′.

### Extracellular vesicle purification

EVs were prepared from the supernatant of H1975 cells by ultra-high-speed centrifugation (Beckman Coulter Optima L-100 XP ultracentrifuge). First, cells were cultured to approximately 70% confluence in cell culture dishes with DMEM containing 10% FBS. The mediun was replaced with serum-free DMEM and cells were cultured for 24 hours, followed by the collection of the cell supernatant and centrifugation at 1500g for 10 minutes to remove cells and debris. The supernatant from multiple dishes of cells was stored in centrifuge tubes. The supernatant was balanced and centrifuged in an ultra-high-speed centrifuge for 90 minutes at 100,000g. The supernatant was then removed, and the remaining precipitate contained the EVs. Next, PBS was added to resuspend the EVs, which were then stored at -80°C until it was needed. The protein concentration of the purified EVs was determined using a BCA Protein Assay Kit (Beyotime, Nantong, China).

### Transmission electron microscopy

The EVs were resuspended in PBS and layered onto carbon-coated electron microscope grids. After incubation at room temperature for 5 minutes, the lattice was negatively stained for 2 minutes with 3% tungstophosphate hydrate. The grids were then examined and photographed using a transmission electron microscope (JEM-1200EX; JEOL Ltd., Tokyo, Japan).

### Lentivirus transfection

YAP shRNA and corresponding scrambled RNA lentiviruses were synthesized and purchased from Genechem Company (Shanghai, China). When the cells were 50-60% confluent, the cells were transfected with YAP shRNA and scrambled RNA lentivirus in 6-well plates according to the manufacturer's instructions. The supernatant containing lentivirus was replaced with complete medium after 12 hours. The shRNA sequences were as follows: YAP 5'-GCAUCUUCGACAGUCUUCUTT-3'; negative control: 5'-UUAUCUAGCUUGGUGGCAGTT-3'.

### Plasmid transfection

H1975 cells at 60-70% confluence were transfected with a myc-YAP plasmid using Lipofectamine 2000 (Invitrogen) and the supernatant was replaced with complete medium after 6 hours according to the manufacturer's protocol. The myc-YAP plasmid and a corresponding control were purchased from Genecopoeia (Germantown, MD).

### Immunofluorescence

The lung adenocarcinoma specimens were fixed with 4% PFA for 24 hours, and then were embedded and cut into sections with a 8-µm thickness. The tissue sections were incubated with anti-YAP and anti-CD31 (Cell Signaling Technology) antibodies at 4°C overnight and were stained with the corresponding fluorescent secondary antibodies at room temperature for 1 h. Then the nuclei were stained using 4, 6 diamidino-2-phenylindole (DAPI) (blue). Three visual fields were randomly selected from each specimen for observation. Then we used ImageJ software for fluorescence analysis.

HUVECs were cocultured with H1975 cells that had been transfected with the myc-YAP plasmid. After 0 h, 0.5 h, 2 h and 6 h, the HUVECs were fixed with 4% PFA, permeabilized, and washed with PBS. Next, the HUVECs were blocked with 3% BSA. Then, the cells were incubated with anti-myc primary antibodies overnight and the corresponding FITC secondary antibodies at room temperature for 1 h. Then, the cellular nuclei were stained using DAPI. The cells were viewed using a Nikon Eclipse Ti confocal laser scanning microscope.

To observe whether EVs derived from H1975 can be internalized by HUVECs, we stained HUVECs using 3, 3-dioctadecyloxacarbocyanine perchlorate (DIO) (green). EVs derived from H1975 were labeled with CM-Dil (red), and then incubated with HUVECs for 2 h. Next, the cells were fixed with 4% PFA, permeabilized and washed, and the cellular nuclei were stained with DAPI. The fluorescence was detected using a confocal laser scanning microscope.

### Protein extraction and western blotting

Total protein was extracted from cells and H1975-EVs in RIPA buffer (Cell Signaling Technology Inc) containing PMSF (Beyotime) and was quantified using a BCA Protein Assay Kit (Beyotime). Thirty-microgram protein samples were separated by 10% SDS-PAGE, transferred to polyvinylidene difluoride (PVDF) membranes (Bio-Rad) and incubated overnight at 4 °C with primary antibodies. The antibodies to human YAP, CD63, GAPDH and anti-rabbit IgG were all purchased from Cell Signaling Technology. The antibodies to human CD81, Alix, Tsg101 were purchased from Abcam. After incubation with goat anti-rabbit IgG secondary antibody at 37 °C for 1 hour, the target proteins were visualized on the PVDF membranes using a Pierce ECL kit and were imaged using a DNR BioImaging System (DNR, Jerusalem, Israel).

### Enzyme-linked immunosorbent assay

The YAP levels in the supernatant of H1975 cells were measured using Quantikine enzyme-linked immunosorbent assay (ELISA) kits according to the manufacturer's protocol (MultiSciences Biotech Co., Ltd., Hangzhou, China). Finally, the signals were measured at a 450 nm wavelength and were plotted according to the numerical values. Each experiment was replicated three times.

### Wound-healing assay

HUVECs were incubated in 6-well plates with complete medium. When the cells were at full confluence as a monolayer, a line of HUVECs was gently and slowly scratched using a 10-µL pipette tip. The detached cells were removed by gently washing twice with PBS. Four groups (DMEM, DMEM containing EVs, DMEM containing shYAP NC-EVs, DMEM containing shYAP-EVs) of culture medium supernatant were added to various plates. The scraped lines were photographed by electron microscopy immediately after scratching and 24 hours later. The wound width was measured using ImageJ software, and the percentage of wound healing was calculated using the following formula: (initial wound size-initial wound size / initial wound size × 100%. The experiment was replicated three times.

### Tube formation assay

Tube formation tests were used to detect the ability of endothelial cells to generate vessels. Matrigel (BD Biosciences, Franklin Lakes, NJ) and DMEM were mixed at a ratio of 1:1. A total of 50 µl of the mixture was added to each well of 96-well plates and was incubated in a cell incubator for 30 minutes. HUVECs (4 × 10^4^) were resuspended in four groups of culture medium supernatant and were seeded in triplicate on the Matrigel-coated wells. HUVECs were incubated at 37 °C for 3 hours. Next, an inverted phase contrast microscope was used to observe the formation of tubules, and the degree of tube formation was analyzed by counting the number of complete tubes.

### Statistical analysis

At least three independent experiments were performed for each assay. All statistical analyses were performed using GraphPad Prism (Version 5.0; La Jolla, CA). All values are expressed as the means ± SD. Statistical significance was determined using the Mann-Whitney test, one-way analysis of variance or two-tailed pearson's correlation analysis. *P*-values less than 0.05 were considered statistically significant.

## Results

### Expression of YAP in lung adenocarcinoma tissue and its correlation with CD31

**Figure 1:**
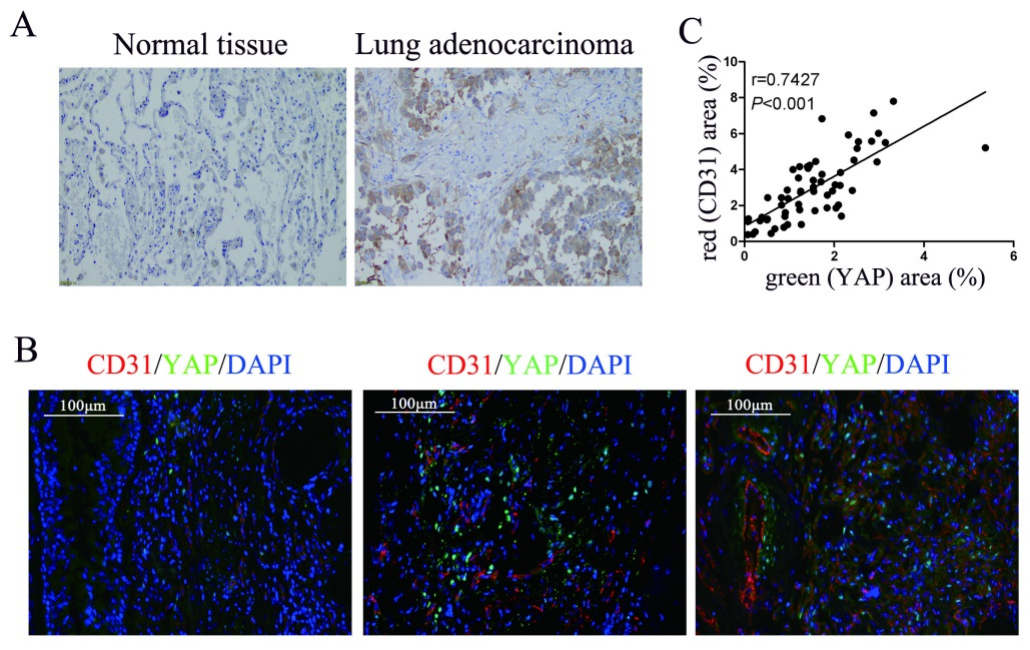
** Correlation between YAP and CD31 expression in human lung adenocarcinoma tissues. (A)** Immunohistochemical detection of YAP in normal lung tissues and lung adenocarcinoma tissues.** (B)** Immunofluorescence detection of YAP and CD31 in lung adenocarcinoma tissues, DAPI was used to visualize the nucleus. The scale bars represent 100 μm. **(C)** YAP and CD31 levels were positively correlated in lung adenocarcinoma tissues (two-tailed pearson's correlation analysis, r=0.7427; *P*<0.001).

To detect the relationship between YAP and angiogenesis in lung adenocarcinoma tissues, we performed immunohistochemical and immunofluorescence tests on surgically resected specimens. First, we detected the expression of YAP in lung adenocarcinoma specimens and normal lung tissues and found that the level of YAP in lung adenocarcinoma was significantly higher than that in normal lung tissue (Fig. 1A). In further experiments, we detected YAP and the vascular endothelial marker CD31 in lung adenocarcinoma tissues by immunofluorescence and found that the expression of CD31 increased with increasing YAP levels in lung adenocarcinoma tissues (Fig. 1B and 1C).

### YAP is highly expressed in lung adenocarcinoma H1975 cells but is expressed at a lower level in normal HUVECs

**Figure 2:**
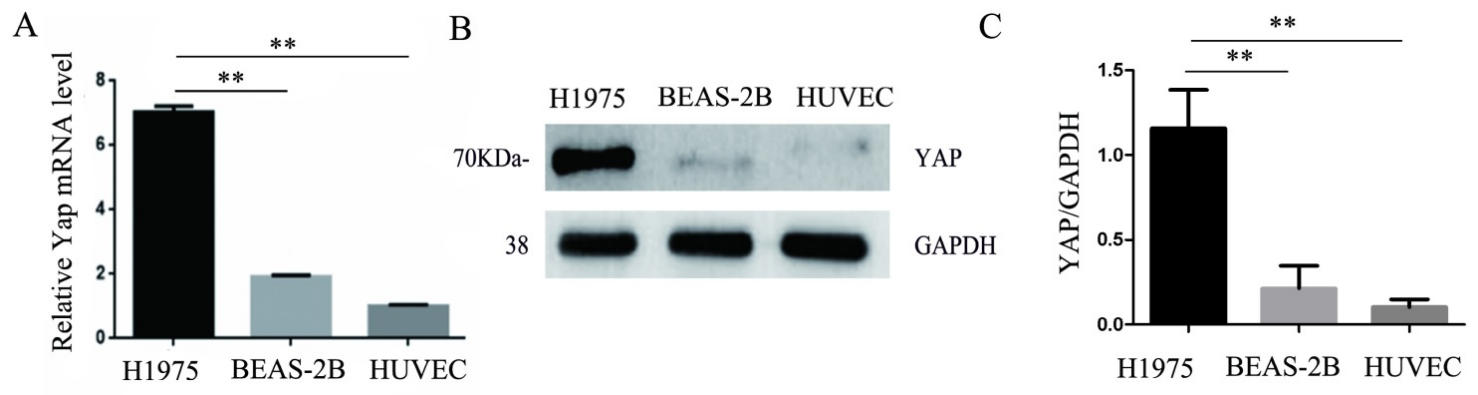
** Expression of YAP in three different cell lines.** (**A**) Real-time quantitative PCR analysis of the YAP mRNA levels in the H1975, BEAS-2B, and HUVEC cell lines. GAPDH was used as an internal reference. The values are presented as the means of three independent experiments. (**B, C**) Expression of YAP in H1975, BEAS-2B and HUVECs, as measured by western blotting. GAPDH was used as an internal reference. **P < 0.01 was considered statistically significant.

Consistent with the results of immunohistochemistry, the expression of YAP in the human lung adenocarcinoma cell line H1975 was higher than that in the normal bronchial epithelial cell line BEAS-2B. Moreover, we found that HUVECs expressed less YAP at the mRNA and protein levels compared to those in the H1975 cells (Fig. 2A-C).

### YAP in H1975 cells affects the expression of YAP in endothelial cells

**Figure 3:**
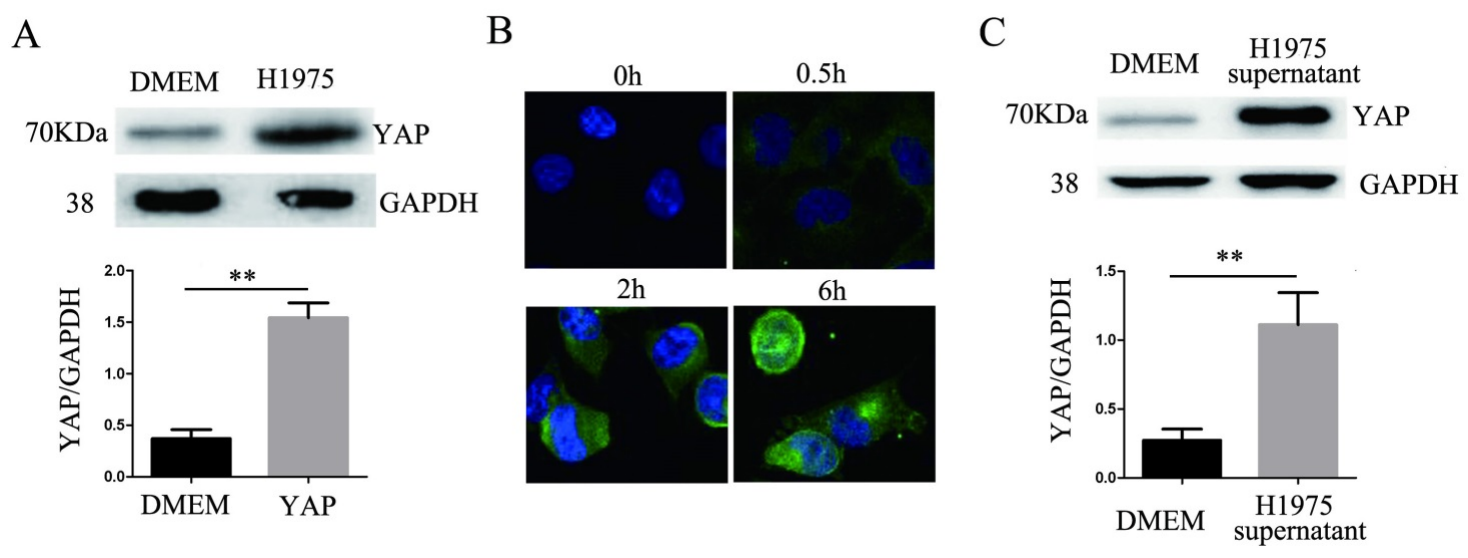
** YAP is transferred from H1975 cells to HUVECs.** (**A**) HUVECs were cocultured with H1975 cells, and the YAP level in HUVECs was detected by western blotting. (**B**) The myc-YAP that was expressed in H1975 was transferred into HUVECs, as demonstrated by immunofluorescence with an anti-myc antibody and a corresponding FITC secondary antibody. DAPI was used to visualize the nucleus. (**C**) HUVECs were cocultured with H1975 cell supernatants, and the level of YAP in HUVECs was detected by western blotting. GAPDH was used as an internal reference. **P < 0.01 was considered statistically significant.

After 24 hours of coculturing H1975 cells and HUVECs, the level of YAP in the HUVECs increased significantly compared to that in the controls (Fig. 3A). HUVECs were cocultured with H1975 cells that had been transfected with the myc-YAP plasmid. The internalization of myc-YAP into HUVECs was visualized by immunofluorescence with an anti-myc antibody and a corresponding FITC secondary antibody. With increasing coculture time, an increasing amount of green fluorescence appeared in HUVECs, indicating that myc-YAP accumulated in HUVECs in a time-dependent manner (Fig. 3B). Similarly, when the H1975 cell supernatant and HUVECs were cocultured for 24 hours, the YAP level in HUVECs also increased significantly compared to that in the controls (Fig. 3C).

### YAP secreted from H1975 cells is transferred to HUVECs via EVs

**Figure 4:**
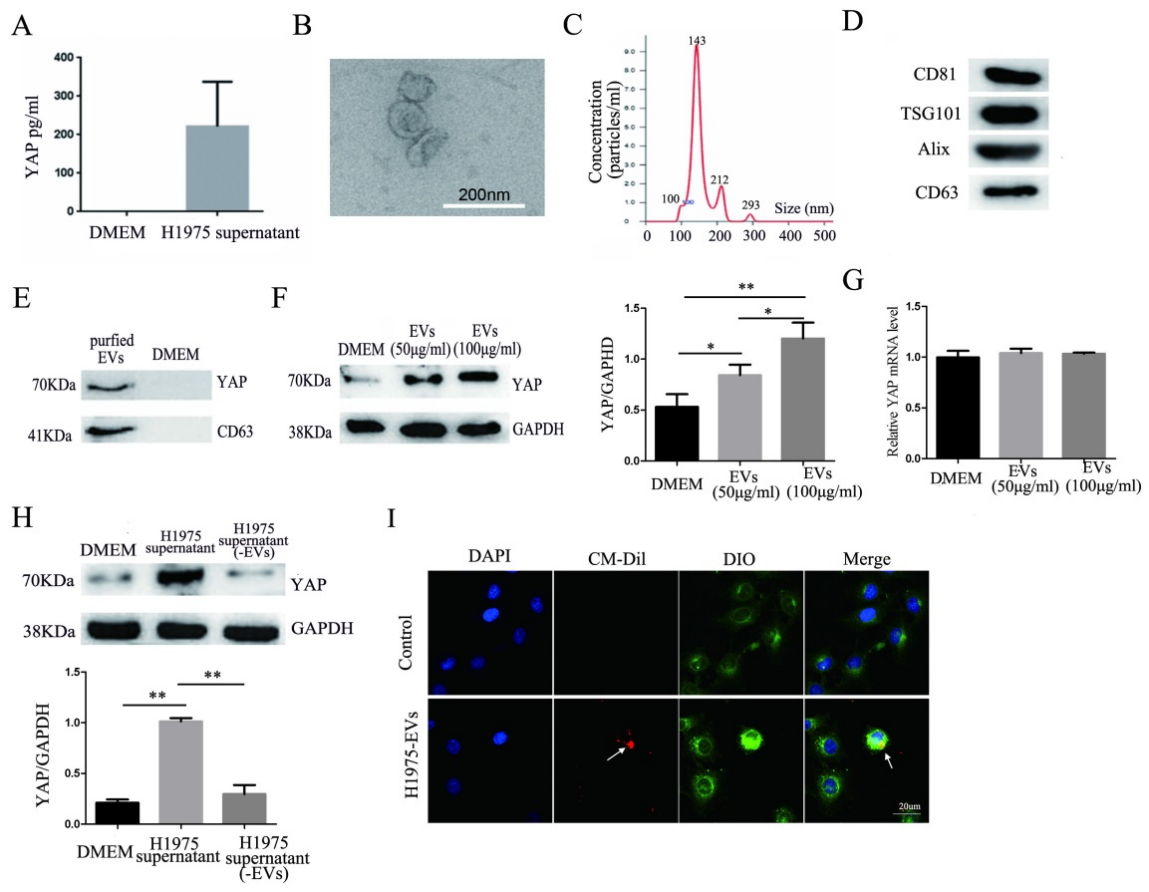
** YAP in H1975 cells is transferred to HUVECs via EVs.** (**A**) ELISA was used to measure the level of YAP in the H1975 supernatant, with pure DMEM used as a control. (**B**) We observed EVs that were isolated from H1975 cell supernatants by TEM. The scale bar represents 200 nm. (**C**) The size distribution of the EVs was examined using NanoSight NS300. (**D**) The EV marker proteins CD81, TSG101, Alix and CD63 were detected by western blotting. (**E**) YAP in the EVs purified from H1975 cell supernatants was detected by western blotting. DMEM was used as a control. (**F**) The level of YAP in HUVECs that were cocultured with EVs at different concentrations was detected by western blotting. GAPDH was used as the internal reference. (**G**) The mRNA level of YAP in HUVECs that were cocultured with H1975 supernatant-purified EVs at different concentrations was detected by real-time quantitative PCR. GAPDH was used as an internal reference. (**H**) HUVECs were cocultured with the H1975 supernatant and EV-free supernatant. The YAP level in HUVECs was detected by western blotting, and GAPDH was used as the internal reference. (**I**) EVs from H1975 cells were labeled with CM-Dil (red) and added to DIO labeled HUVECs for 2 h, and were observed by confocal microscopy. DAPI staining was used to visualize the nuclear areas. The scale bar represents 20 μm. *P < 0.05; **P < 0.01 was considered statistically significant.

The results of an ELISA showed the presence of YAP in the H1975 cell supernatant (Fig. 4A). EVs were isolated and purified from the H1975 cell supernatant. As shown in Fig. 4B-D, the EVs were typical, round-shaped, membranous particles, with sizes ranging from 78 to 331 nm in diameter and with an average diameter of 143 nm. The EVs expressed CD81, TSG101, Alix, and CD63 (markers of EVs).

The presence of YAP in the EVs was also detected by western blotting (Fig. 4E). In addition, H1975 supernatant-purified EVs and HUVECs were cocultured for 24 hours. Western blotting showed that the level of YAP in HUVECs increased with increasing EV concentrations (Fig. 4F). Meanwhile, real-time quantitative PCR showed that there was no significant difference at the mRNA level of YAP among the HUVECs that were cultured with different concentrations of EVs (Fig. 4G). H1975 supernatants and EV-free supernatants were cocultured with HUVECs for 24 hours. The level of YAP in HUVECs was detected by western blotting and showed that the YAP level was significantly higher in HUVECs cocultured with the H1975 supernatant than in HUVECs cocultured with EV-free supernatant or with DMEM (Fig. 4H). To verify whether EVs can be transferred to endothelial cells, we pretreated DIO-labeled HUVECs with CM-Dil-labeled EVs in vitro, and the EVs were shown to fuse with the membranes of the HUVECs (Fig. 4I). These results suggest that YAP can be transported from H1975 cells to HUVECs through EVs, at least partially.

### Interfering with YAP in H1975 cells reduces the expression of YAP in HUVECs

**Figure 5:**
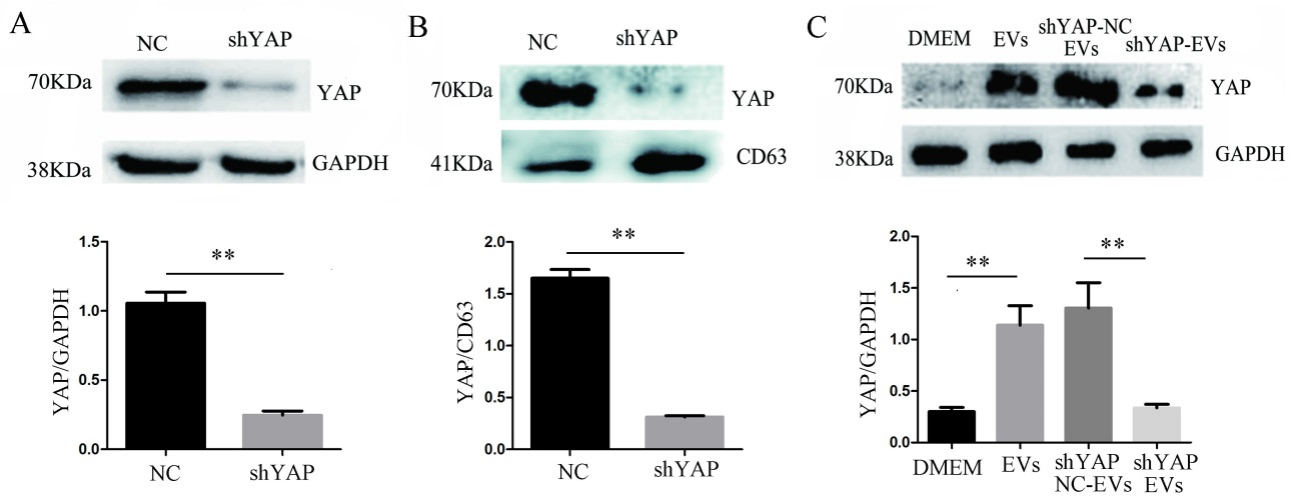
** Interference of YAP expression in H1975 cells affects the expression of YAP in HUVECs.** (**A**) The level of YAP in H1975 cells transfected with shYAP and its NC lentivirus was detected by western blotting, with GAPDH used as an internal reference. (**B**) The level of YAP in EVs was also detected by western blotting, and CD63 was used as the internal reference. (**C**) After the coculture of HUVECs and EVs derived from H1975 that had been transfected with the shYAP and NC lentiviruses, YAP in HUVECs was detected by western blotting. Each experiment was repeated three times. **P < 0.01 was considered statistically significant.

When H1975 cells were transfected with shYAP lentiviruses, we confirmed, by western blotting, that the level of YAP in the cells was significantly reduced compared to that in the controls (Fig. 5A). At the same time, we observed that compared to the controls, the YAP level in the EVs was also decreased (Fig. 5B), indicating that the level of YAP in the EVs was consistent with the level of intracellular YAP protein. After coculturing shYAP-H1975 derived EVs with HUVECs for 24 h, the level of YAP in the HUVECs was also decreased compared to that in the controls (Fig. 5C).

### Effects of YAP on the migration and angiogenesis of HUVECs

**Figure 6:**
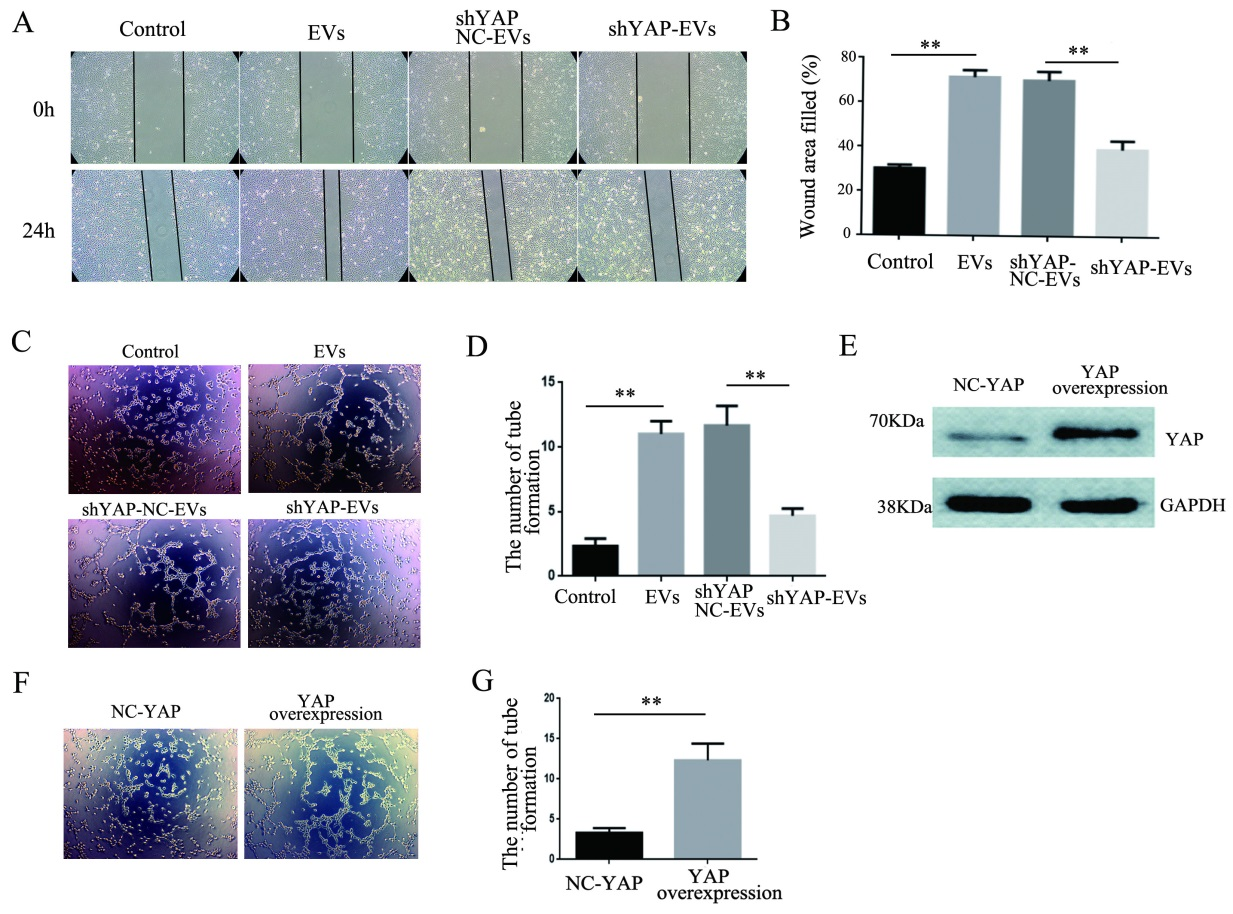
** Effects of YAP derived from EVs on the migration and angiogenesis of HUVECs.** (**A**) Wound-healing assays were performed with HUVECs after coculture with H1975 EVs and H1975 EVs transfected with NC and YAP shRNA lentiviruses and showed that YAP enhanced the migration ability of HUVECs. (**B**) Quantification of the migration ability was performed with HUVECs that were cocultured with four supernatant groups. (**C**) Tube formation tests were performed on four groups: DMEM, H1975 EVs, and H1975 EVs transfected with NC and YAP shRNA lentiviruses. The results showed that YAP enhances the angiogenic ability of HUVECs. (**D**) Quantification of the angiogeic ability of HUVECs that were treated with the four supernatant groups. (**E**) The level of YAP in HUVECs was detected by western blotting after transfection with the YAP overexpression lentivirus. (**F**) Tube formation assays were performed with HUVECs transfected with YAP overexpression and NC lentiviruses. HUVECs with high YAP level showed a higher angiogenic ability. (**G**) Quantification of the angiogenic ability of HUVECs that overexpressed YAP. The values are represented as the means of three independent experiments. **P < 0.01 was considered statistically significant.

To explore the effect of YAP from H1975 cells on HUVECs, wound healing and tube formation assays were performed on HUVECs. The results showed that the migration ability of HUVECs treated with purified shYAP-H1975-derived EVs was significantly lower than that of the control group (Fig. 6A, B). Similarly, the angiogenic ability of HUVECs treated with shYAP-H1975-derived EVs was significantly decreased compared to that of the controls (Fig. 6C, D).

Western blotting showed that the YAP level in HUVECs transfected with YAP overexpression lentiviruses was increased compared to that in the controls (Fig. 6E). Additionally, we found that the angiogenic ability of HUVECs was increased significantly when the YAP level was increased (Fig. 6F, G). These results indicated that the YAP level of HUVECs affects their angiogenesis ability.

## Discussion

Angiogenesis is known to be involved in each stage of cancer, including tumorigenesis 22, 23, progression 24, invasion, and metastasis 25, 26. Angiogenesis is a complex, multistep process with dynamic changes in endothelial cells 27. Endothelial cell function is regulated by many genes 28. Recently, YAP was shown to control endothelial cell function 29. Our study further supports that YAP promotes the angiogenesis of endothelial cells. Additionally, YAP expression was found to be elevated in hepatocellular carcinoma, prostate cancer, colon cancer, ovarian cancer, and breast cancer 30-32. Consistent with previous reports, we found that the expression of YAP in lung adenocarcinoma tissue was also higher than that in normal tissue. Moreover, there is a positive correlation between the YAP level and the vascular endothelial marker protein CD31 level in lung adenocarcinoma tissue. This finding further supports the hypothesis that YAP may promote angiogenesis in cancers.

The interaction between tumor cells and endothelial cells affects endothelial cell function. This communication is generally believed to be how tumors secrete multiple substances that affect other cells in the tumor microenvironment. Indeed, the content of YAP in lung cancer cells is much higher than that in endothelial cells. Based on the increased expression of YAP in endothelial cells after stimulation, whether the tumor cells were cocultured with endothelial cells or stimulated by tumor cell supernatant, we speculate that tumor cells may affect endothelial cell function by secreting YAP.

There are many ways to communicate between cells, such as via ligand-receptor binding 33, membrane-to-membrane contact 34, and the release of soluble mediators [35]and EVs 36. EVs are cell-derived nanoparticles that are increasingly considered to be important mediators of biological signals in physiological and pathological processes 37, 38. EVs can act as the carriers of important signals to other cells, thereby modifying the cell function 36. In this study, we demonstrated that YAP can be secreted into cell culture supernatants in EVs. Interestingly, when the EVs were removed from the tumor cell supernatant before coculturing endothelial cells with the supernatant, the expression of YAP in endothelial cells was significantly decreased, and the stimulation of EVs alone did increase the level of YAP in ECs. These results imply that YAP can be transported to target cells in the form of EVs that are secreted from tumor cells. However, it remains unclear whether there is more soluble YAP (soluble protein) in the free cell supernatant or contained in EVs. Additionally, although we demonstrated that YAP is carried by EVs, it was difficult for us to determine whether YAP was localized on the EV-surface or was inside of the EVs using our current methods and technology.

The transmission of EVs from cancer cells to other cell types has been the subject of intensive studies in recent years. It has become increasingly clear that cancer-derived EVs can exert complex effects on endothelial cells, endothelial cells progenitors, and supporting cells, contributing to vessel formation within tumors 39. Knocking down the expression of YAP in H1975 cells and H1975-EVs reduces the migration and tube formation ability of endothelial cells by reducing the amount of YAP that enters the endothelial cells. This further confirms the possibility of cancer-EV-YAP-endothelial-vascular access.

In conclusion, we demonstrated that YAP is transmitted from lung adenocarcinoma H1975 cells to endothelial cells by EVs to regulate the angiogenesis of endothelial cells, suggesting that EVs may mediate communication among cells in the cancer microenvironment. Further research on this mechanism may lead to deeper discoveries regarding the process of tumor metastasis.

## Abbreviations

EVs: Extracellular Vesicles
HUVECs: Human Umbilical Vein Endothelial Cells
YAP: Yes Kinase-associated Protein
NSCLC: non-small cell lung cancer
LUAD: lung adenocarcinoma
LSCC: lung squamous cell carcinoma
EGFR: epidermal growth factor receptor
